# A longitudinal investigation of psychological morbidity in patients with ovarian cancer

**DOI:** 10.1038/sj.bjc.6604770

**Published:** 2008-11-11

**Authors:** V Gonçalves, G Jayson, N Tarrier

**Affiliations:** 1 Academic Division of Clinical Psychology, School of Psychological Sciences, University of Manchester, 2nd Floor, Zochonis Building, Brunswick Street, Manchester M13 9PL, UK; 2 Department of Medical Oncology, Cancer Research UK and University of Manchester, Christie Hospital NHS Trust, Wilmslow Road, Manchester M20 4BX, UK

**Keywords:** ovarian cancer, anxiety, depression, longitudinal study

## Abstract

Ovarian cancer patients may experience psychological disorders due to the aggressive nature of the illness and treatment. We investigated the presence of psychological disorders longitudinally in women with a new diagnosis of ovarian cancer and the factors that predicted development and maintenance of these disorders. Patients were assessed in a prospective longitudinal study at the beginning of chemotherapy treatment, mid-treatment, end of treatment and 3 months follow-up for depression, anxiety, perceived social support, neuroticism and cognitive strategies to control unwanted thoughts. A total of 121 patients were recruited and 85 patients were assessed at all four time points. Three different longitudinal profiles of anxiety and depression caseness were found: non-cases (never cases), occasional cases (cases on at least one but not all four occasions) and stable cases (cases on all four occasions). Most of the women were occasional cases of anxiety (52%, 44), whereas for depression, the majority of women were non-cases (55%, 47). A subset of patients were stable cases of anxiety (22%, 19). Neuroticism and marital status were significant independent predictors of anxiety caseness profile. Neuroticism and use of anti-depressants were independent predictors of depression caseness profile. Social support was not related to psychological morbidity.

Ovarian cancer is the fourth highest cause of mortality in female cancer patients and the most fatal malignancy of the female genital tract. It has a lifetime risk of approximately 2%. It is uncommon before the age of 40, but the incidence increases with age, reaching a peak in the late 70s. The disease presents, predominantly, at an advanced stage, with a 5-year survival rate of 45%. Less than 20% of tumours are localised at the time of diagnosis (American Cancer Society, 2007).

Ovarian cancer patients may be at high risk of developing psychological morbidity (Hipkins *et al*, 2004); however, the evaluation of psychological morbidity, such as anxiety and depression, has received relatively little attention. Some studies have assessed ovarian cancer patients in samples of all women diagnosed with gynaecological cancer (Cain *et al*, 1983; Andersen *et al*, 1989; Petersen *et al*, 2005). However, as gynaecological cancers have different profiles, the psychological challenges associated with these various malignancies may differ (Rieger, Touyz and Wain, 1998). Published research evaluating ovarian cancer include mostly qualitative studies (Ferrell *et al*, 2002, 2003a, 2003b, 2003c; Howell *et al*, 2003) and quality-of-life studies (Guidozzi, 1993; Anderson, 1994; Kornblith *et al*, 1995; Ersek *et al*, 1997; Borduka-Bevers *et al*, 2000; Lakusta *et al*, 2001). These studies indicate that disruptions to quality of life may occur, which includes significant distress, impairment in physical, vocational, social, familial and sexual functioning. A deterioration in quality of life was evident 2 years after the commencement of treatment (Guidozzi, 1993). Studies assessing the prevalence of psychological distress report rates of 22–47% for anxiety (Kornblith *et al*, 1995; Borduka-Bevers *et al*, 2000; Hipkins *et al*, 2004) and 6–35% for depression (Kornblith *et al*, 1995; Borduka-Bevers *et al*, 2000; Wenzel *et al*, 2002; Hipkins *et al*, 2004; Norton *et al*, 2004). Longitudinal studies addressing the prevalence of psychological distress from diagnosis to follow-up are lacking. Despite some inconsistent findings, demographic factors and medical factors have been associated with the development of psychological distress (Kornblith *et al*, 1995; Borduka-Bevers *et al*, 2000; Hipkins *et al*, 2004; Norton *et al*, 2004). Poor social support has also been associated with the presence of psychological disorders in ovarian cancer patients (Hipkins *et al*, 2004). Little is known regarding personality factors and coping strategies as risk factors of psychological morbidity in ovarian cancer patients.

This study was designed to investigate the presence of psychological disorders in ovarian cancer patients from immediately after diagnosis and referral to a specialist treatment centre and over the period of their treatment and follow-up. We were interested in identifying caseness for psychological disorders and not just symptoms or distress, as this was considered a more important clinical outcome and accurate assessment of significant psychological morbidity. We used a prospective longitudinal design, in which patients were assessed at similar time points during their illness course and treatment. This allowed us to investigate the course of psychological disorders over time and identify those patients in which psychological morbidity was persistent. We hypothesised that (i) three different psychological morbidity profiles would appear across time, which were stable cases (patients who were always caseness for anxiety or depression), occasional cases (cases on some occasions of assessment but not all) and non-cases of anxiety and depression (patients who were never classified as cases); (ii) poor social support, high neuroticism and use of worry as a coping strategy to control unwanted thoughts would be associated with the development and persistence of psychological morbidity.

## Materials and methods

### Participants

Participants were recruited from consecutive new referrals attending the ovarian cancer outpatient clinic of Christie Hospital, Manchester, UK, a specialist cancer centre, subsequent to their diagnosis but before the start of their treatment. Eligible participants were patients with a new diagnosis of ovarian, peritoneal or fallopian tube carcinoma, who provided informed consent, who did not have a pre-existing organic brain disorder or severe mental illness and were able to understand English language. Written consent was obtained from all patients.

Patients with a diagnosis of peritoneal or fallopian tube carcinoma were recruited because they are remarkably similar in their morphology, clinical behaviour and treatment to ovarian cancer (Kosary and Trimble, 2002; Piek *et al*, 2004). Participants were recruited into the study before commencing a first-line platinum-based chemotherapy regimen, conventionally administered every 3 weeks for six cycles (Adams *et al*, 1998). Routine computed tomography scans were performed at the start and at the end of treatment.

### Procedure

New referrals to the ovarian cancer treatment centre were approached to participate in the study at their first appointment at the clinic. Participants who consented to take part in the study completed the assessment measures at four time points: time 1 (T1, first cycle of chemotherapy; beginning of treatment), time 2 (T2, third cycle of chemotherapy; half-way treatment, approximately 6–8 weeks after T1), time 3 (T3, sixth cycle; end of chemotherapy, approximately 15 weeks after T1), and time 4 (T4, 3 months follow-up after cessation of treatment, approximately 27 weeks after T1).

Ethical Committee approval was obtained from the Local Research Ethics Committee before commencement of the study.

### Measures

#### Demographic and clinical information

A structured interview schedule was used to record general demographic and clinical information including age, marital status, employment status and education. Coexisting physical health problems, current mental health problems, current psychological treatment, past mental health problems, past psychological treatment (mental health problems and psychological treatment were assessed as just present or absent), current anti-depressant medication, time since cancer diagnosis and family history of cancer were also assessed. A subjective rating of current subjective physical health was obtained by asking patients to rate their physical health on a 7-point scale (0=extremely ill to 6=well, healthy) (Matthews *et al*, 1999).

#### Medical information

Histological diagnosis, stage of disease according to FIGO (International Federation of Gynecology and Obstetrics), Karnofsky Performance status (KP; Karnofsky and Buchernal, 1949), chemotherapy regimen received and response to chemotherapy treatment (WHO, 1979) were recorded from the clinical notes and from the medical database.

#### Psychological measures

The following standardized psychological measures were used; all have acceptable psychometric properties.

#### Anxiety and depression

The Hospital Anxiety and Depression Scale (HADS; Zigmond and Snaith, 1983) was the primary outcome measure of psychological morbidity, anxiety and depression, assessed at different time periods. It was administered at all time points. The HADS is a 14-item scale that measures anxiety (seven items) and depressive symptoms (seven items). For the identification of clinical cases, the cutoff scores recommended for both subscales are a score of 8 or above indicating a possible case and a score of 11 or above indicating a probable case of anxiety or depression. A score of 0–7 for either subscale is considered within the normal range. In this study, scores of 8 or above were considered as cases of anxiety and depression at that assessment point.

#### Perceived social support

The Social Support Inventory (SSI; Holeva, 1998) assessed the effect of perceived criticism and perceived social support on the development and maintenance of psychological disorders. It was completed at time 1. It includes five items. Two items refer to perceived criticism of, and from, significant others and three items refer to social support. The items are reverse-scored, so high scores indicate an absence of criticism but poor social support. The SSI has previously been used with ovarian cancer patients and found to predict psychological morbidity (Hipkins *et al*, 2004). The SSI has good psychometric properties: internal consistency (Cronbach alpha: social support 0.83; perceived criticism 0.67) and 4-week test–retest reliability (social support *r*=0.55; perceived criticism *r*=0.79).

#### Coping strategies to control unwanted thoughts

The Thought Control Questionnaire (TCQ; Wells and Davies, 1994) assessed the use of worry and other coping strategies to control unwanted thoughts on the development and maintenance of psychological morbidity. It was completed at time 1. It comprises 30 items, which include five subscales that assess five strategies to control intrusive thoughts: distraction, punishment, reappraisal, social control and worry. It has previously been demonstrated to be predictive of the development of psychological disorders in trauma patients (Holeva *et al*, 2001). The TCQ has good psychometric properties with a Cronbach alpha of between 0.64 and 0.79 for the five scales and a test–retest reliability of between *r*=0.68 and *r*=0.83).

#### Neuroticism

The Revised Eysenck Personality Questionnaire (EPQ-R; Eysenck and Eysenck, 1991) evaluated the effect of neuroticism on the development and maintenance of psychological morbidity. It was completed at time 1. In this study, only the Neuroticism subscale was employed. Persons scoring high in neuroticism are described as anxious, worrying, moody and depressed. Neuroticism is significantly associated with the development of psychological disorders after trauma (Holeva and Tarrier, 2001).

### Statistical analysis

Data were analysed using the SPSS Version 13.0. Descriptive statistics were used to analyse the sample characteristics and psychological measures.

Patients were divided into three groups according to their profile of the occurrence and persistence of anxiety and depressive disorders across time. For both anxiety and depression separately, the following classifications were made: (1) patients who were never cases on any of the four assessment time points – ‘stable non-cases’; (2) patients who were cases on at least one assessment but not all four assessments – ‘occasional cases’; (3) patients who were cases on all four assessments – ‘stable cases’. Cases comprised of scoring 8 or above in the HADS anxiety or depression scale at that assessment time point.

Non-parametric statistical tests were used to compare measures that were categorical and where measures significantly departed from normal; otherwise, parametric statistics were used for group comparisons. Tukey's honestly significant differences (HSD) test was used for all *post hoc* analyses. Multinomial Logistic Regression analysis was performed to estimate adjusted odds ratios (ORs) and 95% confidence intervals (CIs) for confounding variables. This method calculates ORs for a dependent variable with more than two categories, evaluating the effect of each explanatory variable. This analysis was performed to predict the three-category-dependent variable: ‘stable non-cases’, ‘occasional cases’ and ‘stable cases’ of anxiety. Binary Logistic Regression was used to predict the two-category-dependent variable: ‘stable non-cases’ and ‘cases’ of depression (combining occasional and stable cases). This was carried out using Stepwise Forward (Wald) model. Patient characteristics, medical and psychological variables identified in the univariate analysis with a *P*=0.1 (significance at a 10% level) entered the first model of the Multinomial Logistic Regression and the Binary Logistic Regression model. Statistical significance was set at *P*⩽0.05.

## Results

### Participants

From 148 patients who met the inclusion criteria, 25 refused participation and 2 provided informed consent but failed to return the questionnaires. The most common reasons for refusing to participate in the study were that the patient was not interested in the study or did not want to be reminded about their cancer. A total of 121 patients participated in the study and completed the measures for at least one time point, with 118 completing assessments at T1, 106 at T2, 100 at T3 and 95 at T4. Eighty-five patients completed all four assessment points. Eleven patients were assessed only once, 7 only on two occasions, 17 on three occasions and 86 on all four occasions. Eleven patients died during the duration of the study. Table 1 shows the percentages of non-cases and cases of anxiety and depression at each assessment point measured by the HADS. The majority of the sample scored in the clinical range of anxiety. The highest percentage of probable cases of anxiety (scores >11) occurred at time 1. Most of the patients were not cases of depression. The highest percentage of patients scoring in the clinical range of depression occurred at time 1. Tables 3 and 4 present the patients' characteristics and results of the psychological measures for the complete data set (*n*=121).

Of the 121 patients, 36 were excluded from the present longitudinal analysis due to incomplete data. Comparisons were made, on all variables, between the 85 patients with a complete data set and the 36 with incomplete data. Owing to the large number of comparisons, significance was set at 1%. There were no differences on demographic characteristics, psychological variables, time since cancer diagnosis, treatment type, stage of disease and type of diagnosis. Those with complete data had significantly higher KP scores (*U*=1012.000, *P*=0.008; medians −80 and 70, respectively) and a better response to treatment (*χ*^2^ (2)=9.25, *P*=0.01). It is possible that those who did not complete assessments did so due to the severity of their illness.

The longitudinal pattern of anxiety and depression was examined for 85 patients who completed the measures at all the four assessment points. Patients were allocated to profile groups depending on the occurrence and persistence of anxiety and depression: (1) ‘stable non-cases’, (2) ‘occasional cases’, and (3) ‘stable cases’. Table 2 shows the number of patients in each profile group according to their anxiety and depression profiles. The most prevalent profile of anxiety was ‘occasional cases’ (52%, 44), indicating that clinical anxiety was frequently intermittent, whereas ‘stable non-cases’ was the most common profile of depression (55%, 47), indicating that clinical depression was not prevalent. The distribution of patients’ characteristics and medical data according to the profile groups is presented in Table 3 and psychological variables are presented in Table 4.

### Anxiety

Comparisons of three anxiety profile groups were made on all characteristic and medical data. There were statistically significant differences among the groups with respect to age (*F*(2, 82)=3.03; *P*=0.054). *Post hoc* analysis indicated that patients in stable cases were younger than those in stable non-cases. Groups also differed regarding marital status (*χ*^2^ (2)=6.11; *P*=0.047) and length of time since cancer diagnosis (*F*(2, 82)=5.43; *P*=0.006). The proportion of married women was greater in stable cases. Compared with the other two groups, patients in stable non-cases had a longer length of time since cancer diagnosis when compared to those in other groups.

The three groups differed significantly regarding neuroticism (*F*(2, 79)=19.04; *P*<0.001). Non-cases had the lowest scores of neuroticism, whereas stable cases exhibited the highest scores of neuroticism. Higher levels of neuroticism were associated with higher rates of anxiety, which tended to be persistent (stable cases). The three groups also varied significantly in the use of worry to control unwanted thoughts (*F*(2, 78)=3.94; *P*=0.024), with non-cases having significantly lower worry scores and stable cases the highest.

### Depression

Occasional cases and stable cases were combined due to a low number of patients in stable cases. This resulted in two groups of patients: ‘non-cases’ (*n*=47) and ‘cases’ (*n*=38). Comparisons of the two depression profile groups indicated statistically significant differences between the two groups with respect to subjective physical health rating scores (*U*=666.500, *P*=0.038), use of anti-depressants (*χ*^2^ (1)=8.91, *P*=0.003), length of time since cancer diagnosis (*t*(83)=2.87; *P*=0.005), stage of disease (*U*=669.500, *P*=0.027) and KP (*U*=601.500, *P*=0.018). Non-cases self-rated their health status as better compared with cases (medians: non-cases 5; cases 4). Significantly more patients in the cases group had taken anti-depressants. There was a longer time since diagnosis in non-cases (mean=61 days) than in cases (mean=45 days). Patients in the cases group had more advanced FIGO stage (stage III or IV) and lower KP scores than those in non-cases. Patients in the cases group reported higher neuroticism (*t*(80)=−4.05, *P*<0.001) and used more worry as a coping strategy to control unwanted thoughts (*t*(79)=−2.33, *P*=0.022) than non-cases.

### Predictors of persistent psychological disorder

#### Predictors of anxiety profile group

Age, marital status, education, length of time since cancer diagnosis, neuroticism, worry and punishment entered the first model of the Multinomial Logistic Regression to identify significant independent predictors of anxiety profile groups. The overall statistics for the final equation were *χ*^2^ (4)=38.53, *P*<0.001. Marital status (*P*=0.041) and neuroticism (*P*<0.001) were overall significant and independent predictors of anxiety profile group. Married women (OR=9.38; 95% CI (1.2, 73.36) and those with greater neuroticism (OR=1.59; 95% CI (1.3, 1.95) were at higher risk of being in group 3 (stable cases) compared with no caseness of anxiety. The risk of being a stable case increases by 9.38 if the patient was married compared with not being married and by 1.59 for every unit increase in neuroticism.

#### Predictors of depression profile group

Length of time since cancer diagnosis, KP, stage of disease, subjective physical health rating, current mental health problems, use of anti-depressants, neuroticism, distraction, worry and social control entered the model of the Binary Logistic Regression. The overall statistics for the final equation were *χ*^2^ (2)=17.89, *P*<0.0001. Neuroticism (*P*=0.005) and use of anti-depressants (*P*=0.027) were significant and independent predictors of depression profile group. Patients who used anti-depressants (OR=5.073; 95% CI (1.2, 21.3)) and had higher neuroticism scores (OR=1.17; 95% CI (1.05, 1.3)) were more likely present in the cases group. The likelihood of being a case increased by 5.073 if the patient used anti-depressants and by 1.17 for every unit increase in neuroticism scores.

## Discussion

Our study is the first to assess anxiety and depression in patients with ovarian cancer prospectively and longitudinally over the illness course and treatment. We approached consecutive referrals to a specialist cancer centre of patients who were new cases of ovarian cancer before the commencement of their treatment regimen. This allowed us to assess psychological morbidity over a standard treatment regimen and time period. Thus, avoiding as much as possible the heterogeneity of patients and timing that potentially confounds psychological research in cancer patients. Secondly, we assessed patients over four assessment points so as to investigate the persistence of psychological disorder, thus avoiding the difficulties inherent in cross-sectional studies. We have also assessed ‘cases’ of anxiety and depression rather than symptoms so as to identify clinically significant levels of psychological morbidity rather than just distress, which may be understandable, transitory and less clinically important. Our results support previous findings that anxiety and depression are present after a recent diagnosis of ovarian cancer, and during the treatment of the disease and the follow-up period (Newport and Nemeroff, 1998; Williams and Dale, 2006). We investigated the persistence of psychological disorders by studying the individual variability of anxiety and depression caseness across time. A large number of patients were cases of anxiety and depression on some occasions, although in many this was a temporary condition. Transitory psychological disorders may occur at various stages of the illness and its treatment, but once the crises fade, the disorders remit (Massie and Popkin, 1998). In general, it seems that the majority of women were anxious to a significant degree at some point during their treatment and follow-up. Anxiety is a response to threat, and the diagnosis and treatment may constitute a threat for many patients. The majority of women adjust to their illness (Andersen *et al*, 1989; Hamilton, 1999), but a proportion of patients do develop a persistent psychological disorder, which is more likely to be anxiety. Persistent clinical depression is relatively uncommon.

Marital status and neuroticism were independent predictors of anxiety caseness, whereas use of anti-depressants and neuroticism were the best predictors of depression caseness. Previous studies have shown equivocal results between marital status and psychological distress (Harrison and Maguire, 1994; Mundy *et al*, 2000; Zabora *et al*, 2001). Some consequences associated with ovarian cancer may affect married women more directly, such as having young children and sexual problems (Howell *et al*, 2003). Unsurprisingly, and consistent with previous studies (Costanzo *et al*, 2007), women who used anti-depressants were more likely to be depressed. The use of anti-depressants may indicate that women presented levels of psychological symptoms that led their physician to prescribe medication. Although our study failed to find associations between current and past psychiatric history, earlier studies have found previous psychiatric history to be a predisposing factor to psychological disorders in cancer patients (Berard *et al*, 1998; Costanzo *et al*, 2007).

In this study, neuroticism was associated with persistent psychological morbidity. This is a consistent finding in the general literature (Ormel and Wohlfarth, 1991; Clark *et al*, 1994; Holeva and Tarrier, 2001) and in cancer patients, in particular (Ranchor *et al*, 2002; Millar *et al*, 2005). An individual with high neuroticism experiences more worries, uncertainties, anxiety, depression, and psychosomatic symptoms and has poor coping under stress (Eysenck and Eysenck, 1991). Thus, it would not be unexpected that higher neuroticism would be associated with anxiety and depression, as all of these elements are characteristics of these disorders. High scores on neuroticism in the early stages of cancer could well increase vulnerability to these personality characteristics escalating to full symptoms when exposed to the threat and uncertainty of cancer treatment.

Contrary to our prediction, perceived social support was not associated with psychological morbidity. These results were surprising, as social support has been identified previously as an important factor associated with psychological distress and disorders in patients with ovarian cancer (Nordin *et al*, 2001; Hipkins *et al*, 2004; Norton *et al*, 2005). This finding may reflect the nature of the sample recruited. Patients in our study seemed to have very good social support and relationships with significant others so that there was little variance in the measure, suggesting that ovarian cancer may have brought the patient closer to their significant others (Hamilton, 1999) or that these patients already had supportive significant others before the diagnosis.

The use of worry as a strategy to cope with unwanted thoughts was significantly associated with anxiety and depression over time. Worry relates to persistent rumination in an attempt at problem solving (Matthews and Wells, 1996). Our results confirm that the worry control strategy seems to be inappropriate for controlling intrusive thoughts. This is consistent with studies showing that measures of anxiety and obsessive-compulsive symptoms were related to worry (Wells and Davies, 1994), and individuals who recovered from posttraumatic stress disorder and/or depression were less likely to use worry to control unwanted thoughts than those who did not (Reynolds and Wells, 1999). Worry as a coping strategy inhibits emotional processing by draining the attentional resources. By diverting attention away from the cognitive operations necessary to support emotional processing, it promotes the development and maintenance of emotional vulnerability (Wells and Matthews, 1996).

Our results also showed that women who were persistent cases of anxiety were younger than women that were not cases, and there was a trend towards younger women being persistent cases of depression. This is consistent with previous findings in ovarian cancer patients (Borduka-Bevers *et al*, 2000; Hipkins *et al*, 2004; Norton *et al*, 2004). For these women, the consequences of, and difficulty in adjusting to, ovarian cancer may be greater because of disruption to family or career plans and impairment of sexual functioning. The management of ovarian cancer may induce premature menopause and the loss of childbearing capacity. Furthermore, some women have young children whom they fear they may not see them grow up, which constitutes an additional source of distress.

Lower subjective physical health ratings were significantly associated with caseness of depression. This result agrees with that of our earlier study (Hipkins *et al*, 2004). It is possible that patients with higher psychological morbidity report more physical symptoms. Psychological disorders may increase sensitivity to physical health and focus attention on physical sensations, which as a result become amplified. Our findings also indicate that women who had more recently been diagnosed with ovarian cancer present higher psychological morbidity (Norton *et al*, 2004). It may be that these women remain acutely distressed and have not had enough time to adjust to their diagnosis. Although the association between medical factors and psychological morbidity in ovarian cancer patients is controversial, our results support the view that women with poor performance status and advanced stage of disease presented higher levels of depression (Kornblith *et al*, 1995; Borduka-Bevers *et al*, 2000; Norton *et al*, 2004). In agreement with Hipkins *et al* (2004), response to treatment and type of chemotherapy regimen were not associated with psychological morbidity.

There are limitations to this study. First, we analysed a sample that was representative of ovarian cancer patients in the UK regarding disease stage and age of diagnosis (Incidence of Cancer in UK, 2004). Nevertheless, the results may not generalise to women of different ethnic background or socioeconomic status and for those with other types of cancer. Second, we followed patients for only 3 months after the end of chemotherapy, and levels of psychological morbidity after this time remain unknown. Third, results showed that women scoring high in neuroticism and worry were more likely to be persistent cases of psychological disorders. This may suggest that these measures obtained at time 1 predicted subsequent anxiety and depression. However, it can be argued that these women were more severely anxious and depressed at time 1 because exposure to such stressful situations caused changes in personality and coping skills. Last, we aimed to assess a cohort of patients at four different time points over the 6-month period of their treatment and follow-up. We were successful in collecting complete data from over 70% of the cohort. But there were missing data and these were unlikely to have been missing at random and may have introduced some bias. There may have been pragmatic reasons why some assessments were not complete or the patient's illness could have been influential. Eleven patients died while in the study.

In practical terms, our results support the need of routine and repetitive screening for identification of persistent psychological morbidity and the provision of adequate and appropriate psychological management. There is a question as to whether scarce resources for psychological treatment should be used to target only those whose psychological disorders persist, or should be used more generally to target early in treatment to prevent persistent morbidity or whether all, even transitory, morbidity is a legitimate treatment target. Furthermore, whether screening for risk factors based on predictive associations, such as high levels of neuroticism or ineffective coping strategies, would identify those at high risk of persistent morbidity and serve as treatment candidates. At least in depression, there appears to be an argument for the use of efficacious psychological treatments, such as cognitive behaviour therapy, as the use of anti-depressants is associated with more severe psychological morbidity so that treatment response can be assumed to be incomplete.

## Figures and Tables

**Table 1:** The percentages of patients who scored as a non-case (<7), as a possible case (8–10), and as a probable case (>11) of anxiety and depression at different time points for the all samples (ie, all patients assessed at that time point) and for the patients with complete data set (*N*=85)

	**Time 1**		**Time 2**		**Time 3**		**Time 4**	
	**All sample**	**Complete data set**	**All sample**	**Complete data set**	**All sample**	**Complete data set**	**All sample**	**Complete data set**
**HADS**	***n* (%) (*N*=118)**	***n* (%) (*N*=85)**	***n* (%) (*N*=106)**	***n* (%) (*N*=85)**	***n* (%) (*N*=100)**	***n* (%) (*N*=85)**	***n* (%) (*N*=95)**	***n* (%) (*N*=85)**
*Anxiety*								
Non-case (cutoff ⩽7)	53 (45)	42 (49)	56 (53)	46 (54)	49 (49)	43 (51)	44 (46)	41 (48)
Possible case (cutoff ⩾8 to ⩽10)	27 (22)	18 (21)	26 (25)	21 (25)	27 (27)	22 (26)	28 (30)	25 (29)
Probable case (cutoff ⩾11)	38 (31)	25 (29)	24 (23)	18 (21)	24 (24)	20 (24)	23 (24)	19 (22)
								
*Depression*								
Non-case (cutoff ⩽7)	82 (70)	60 (71)	79 (75)	65 (77)	77 (77)	65 (77)	71 (75)	65 (77)
Possible case (cutoff ⩾8 to ⩽10)	23 (20)	16 (19)	15 (14)	8 (9)	12 (12)	10 (12)	12 (13)	11 (13)
Probable case (cutoff ⩾11)	13 (11)	9 (11)	12 (11)	12 (14)	11 (11)	10 (12)	12 (13)	9 (11)

HADS=Hospital Anxiety and Depression Scale

*n*=number of patients who were cases or not.

Time 1=beginning of treatment; time 2=half-way treatment; time 3=end of treatment; time 4: 3-month follow-up.

**Table 2:** Number of patients in each group according to their profile of anxiety and depression across time (*N*=85)

**HADS**	***n***	**%**
*Anxiety*		
Group 1 (stable non-cases)	22	26
Group 2 (occasional cases)	44	52
Group 3 (stable cases)	19	22
		
*Depression*		
Group 1 (stable non-cases)	47	55
Group 2^a^ (occasional cases)	33	38
Group 3^a^ (stable cases)	5	6

HADS=Hospital Anxiety and Depression Scale.

*n*=number of patients.

a Groups 2 and 3 were combined for further analysis due to the low number of patients in group 3.

**Table 3:** Distribution of patients' characteristics for the total sample (*n*=121), for the anxiety profile groups (*n*=85), and for the depression profile groups (*n*=85)

		**Anxiety profile groups**			**Depression profile groups**	
**Patients' characteristics**	**Total sample, *n*=121**	**Group 1 (stable non-cases), *n*=22**	**Group 2 (occasional cases), *n*=44**	**Group 3 (stable cases), *n*=19**	**Group 1 (occasional non-cases), *n*=47**	**Group 2 (cases), *n*=38**
Mean age in years (s.d.)^a^	61 (12)	64 (9)	60 (12)	56 (8)	61 (10)	59 (12)
						
*Marital status:* n (%)a,^b^						
Married	77 (64)	12 (54.5)	28 (63.6)	17 (89.5)	31 (66.0)	26 (68.4)
Not married	44 (37)	10 (45.5)	16 (36.4)	2 (10.5)	16 (34.0)	12 (31.6)
						
*Education:* n (%)						
No exams taken	56 (46)	6 (27.3)	26 (59.1)	8 (42.1)	22 (46.8)	18 (47.4)
O-levels	30 (25)	6 (27.3)	8 (18.1)	4 (21.1)	9 (19.2)	9 (23.7)
A-levels	12 (10)	4 (18.1)	1 (2.3)	3 (15.7)	4 (8.5)	4 (10.5)
Higher education	23 (19)	6 (27.3)	9 (20.5)	4 (21.1)	12 (25.5)	7 (18.4)
						
*Employment status:* n (%)						
Not working	87 (72)	16 (72.7)	28 (63.6)	14 (73.7)	16 (34.0)	11 (28.9)
Working	34 (28)	6 (27.3)	16 (36.4)	5 (26.3)	31 (66.0)	27 (71.1)
						
Median subjective physical health rating (range)^c^	4 (0–6)	4 (3–6)	4 (2–6)	5 (3–6)	5 (3–6)	4 (2–6)
Other physical health problems: *n* (%)	57 (47)	9 (40.9)	20 (45.4)	8 (42.1)	18 (38.3)	18 (47.4)
Current mental health problems: *n* (%)	17 (14)	2 (9.1)	5 (11.3)	3 (15.7)	3 (6.4)	7 (18.4)
History of mental health problems: *n* (%)	28 (23)	6 (27.3)	11 (25.0)	7 (36.8)	13 (27.7)	11 (28.9)
Current psychological help: *n* (%)	2 (2)	1 (4.5)	0	1 (5.3)	1 (2.1)	1 (2.7)
History of past psychological help: *n* (%)	14 (12)	3 (13.6)	4 (9.1)	4 (21.1)	7 (14.9)	4 (10.5)
Use of anti-depressants: *n* (%)^c^	19 (16)	2 (9.1)	8 (18.1)	6 (31.6)	3 (6.4)	13 (34.2)
Family history of cancer: *n* (%)	75 (62)	15 (68.2)	27 (61.4)	14 (73.7)	31 (66.0)	25 (65.8)
Mean time since cancer diagnosis in days (s.d.)a,^c^	53 (25)	69 (37)	48 (22)	49 (14)	61 (31)	45 (18)
						
*Diagnosis:* n (%)^d^						
Ovarian cancer	106 (88)	22 (100)	39 (88.6)	16 (84.3)	44 (93.6)	33 (86.8)
Primary peritoneal cancer	11 (9)	0	3 (6.8)	3 (15.7)	2 (4.3)	4 (10.5)
Fallopian tube carcinoma	4 (3)	0	2 (4.6)	0	1 (2.1)	1 (2.7)
						
*Stage of disease:* n (%)^c^						
Stage I	29 (24)	7 (31.9)	10 (22.7)	6 (31.6)	16 (34.0)	7 (18.4)
Stage II	4 (3)	3 (13.6)	0	1 (5.3)	3 (6.4)	1 (2.7)
Stage III	67 (55)	9 (40.9)	30 (68.2)	9 (47.4)	25 (53.2)	23 (60.5)
Stage IV	21 (17)	3 (13.6)	4 (9.1)	3 (15.7)	3 (6.4)	7 (18.4)
						
Median Karnofsky performance status scale value (range)^c^^e^^f^^g^	80 (40–100)	80 (40–100)	80 (40–100)	80 (50–90)	80 (50–100)	80 (40–90)
						
*Chemotherapy regimen:* n (%)						
Carboplatin-based	70 (58)	12 (54.5)	21 (47.7)	11 (57.9)	23 (48.9)	21 (55.3)
Other regimens	51 (42)	10 (45.5)	23 (52.3)	8 (42.1)	24 (51.1)	17 (44.7)
						
*Response to treatment:* n (%)^h^^i^^j^						
No measurable disease	28 (25)	8 (40.0)	8 (18.6)	7 (38.8)	16(37.2)	7 (18.4)
Complete response	24 (22)	5 (25.0)	10 (23.2)	5 (27.8)	11 (25.6)	9 (23.7)
Partial response	24 (22)	4 (20.0)	13 (30.2)	2 (11.1)	9 (20.9)	10 (26.3)
Stable disease	17 (15)	2 (10.0)	6 (14.0)	3 (16.7)	4 (9.3)	7 (18.4)
Progressive disease	18 (16)	1 (5.0)	6 (14.0)	1 (5.6)	3 (7.0)	5 (13.2)

*n*=number of patients; s.d.=standard deviation.

a Indicates statistically significant differences in the anxiety profile groups.

b Grouping of categories was employed because frequencies were less than 5.

c Indicates statistically significant differences in the depression profile groups.

d Grouping of categories was used because frequencies were less than 5.

e There were no available data for three patients in the total sample.

f There were no available data for two patients, one from group 1 and one from group 2 in the anxiety profile groups.

g There were no available data for two patients, one from group 1 and one from group 2 in the depression profile groups.

h There were no available data for 10 patients in the total sample.

i Grouping of categories were used because frequencies were less than 5. There were no available data for two patients from group 1, one patient from group 2 and one patient from group 3 in the anxiety profile groups.

j Grouping of categories were used because frequencies were less than 5. There was no available data for four patients from group 1 in the depression profile groups.

**Table 4:** Descriptive statistics for the psychological variables for the total sample, for the anxiety profile groups, and for the depression profile groups

		**Anxiety profile groups**			**Depression profile groups**	
**Psychological variables**	**Total sample**	**Group 1 (stable non-cases)**	**Group 2 (occasional cases)**	**Group 3 (stable cases)**	**Group 1 (stable non-cases)**	**Group 2 (cases)**
*Neuroticism* a, b						
*n*	114	21	43	18	45	37
Mean (s.d.)	10.21 (5.56)	5.38 (4.82)	10.07 (4.39)	14.5 (4.9)	7.78 (5.14)	12.35 (5.02)
						
*Perceived criticism* c						
*n*	118	21	44	19	46	38
Median (range)	18.8 (0.3–20)	19.5 (9.8–20)	19.3 (0.93–20)	14.1 (0.3–20)	19.26 (0.28–20)	19.21 (6.08–20)
						
*Social support* c						
*n*	119	22	44	19	47	38
Median (range)	0.54 (0–28.9)	0.14 (0–4.5)	0.72 (0.4 -15.6)	0.45 (0–10.6)	0.36 (0–15.63)	0.63 (0–10.8)
						
*Distraction*						
*n*	115	21	42	18	45	36
Mean (s.d.)	16.25 (3.82)	16.95 (4.67)	16.21 (3.84)	15.22 (3.69)	16.89 (3.69)	15.31 (4.32)
						
*Punishment* c						
*n*	114	21	42	18	45	36
Median (range)	9 (6–18)	9 (6–16)	9 (6–16)	10 (7–16)	9 (6–16)	9 (6–16)
						
*Re-Appraisal*						
*n*	115	22	42	18	45	36
Mean (s.d.)	11.43 (3.30)	11.67 (3.68)	11.55 (3.41)	11.44 (3.29)	11.62 (3.62)	11.47 (3.18)
						
*Worry* a, b, d						
*n*	113	22	42	18	45	36
Mean (s.d.)	10.19 (3.20)	8.71 (2.72)	10.50 (3.05)	11.44 (3.78)	9.51 (2.90)	11.17 (3.49)
Median (range)	10.00 (6–20)	8 (6–17)	10 (6–20)	14 (6–23)	9 (6–17)	10 (6–20)
						
*Social control* e						
*n*	114	22	42	18	45	36
Mean (s.d).	13.35 (3.39)	13.81 (1.66)	13.45 (3.83)	13 (4.39)	13.96 (2.82)	12.81 (4.19)
Median (range)	14.00 (6–23)	14 (9–16)	14 (6–23)	12.5 (6–20)	15 (7–20)	13 (6–23)

*n*=number of patients; s.d.=standard deviation.

a Indicates statistically significant differences in the anxiety profile groups.

b Indicates statistically significant differences in the depression profile groups.

c The data were not normally distributed for these variables in the total sample and in the anxiety and depression profile groups; therefore, the median and range are displayed.

d The data were not normally distributed for this variable in the total sample; therefore, the median and range are also displayed.

e The data were not normally distributed for this variable in the anxiety profile groups; therefore, the median and range are also displayed.
